# *Arcobacter* Species in Humans^1^

**DOI:** 10.3201/eid1010.040241

**Published:** 2004-10

**Authors:** Olivier Vandenberg, Anne Dediste, Kurt Houf, Sandra Ibekwem, Hichem Souayah, Sammy Cadranel, Nicole Douat, G. Zissis, J.-P. Butzler, P. Vandamme

**Affiliations:** * Saint-Pierre University Hospital, Brussels, Belgium;; † Free University of Brussels, Brussels, Belgium;; ‡ Ghent University, Ghent, Belgium;; § Vrije Universiteit Brussel, Brussels, Belgium;; ¶ Queen Fabiola University Hospital, Brussels, Belgium;; # Brugmann University Hospital, Brussels, Belgium;; ** Queen Fabiola University Hospital, Brussels, Belgium

**Keywords:** *Arcobacter*, *Campylobacter*, diarrhea, case-control, clinical, dispatch

## Abstract

During an 8-year study period, *Arcobacter butzleri* was the fourth most common *Campylobacter*-like organism isolated from 67,599 stool specimens. Our observations suggest that *A. butzleri* displays microbiologic and clinical features similar to those of *Campylobacter jejuni*; however, *A. butzleri* is more frequently associated with a persistent, watery diarrhea.

*Campylobacter* is the most common cause of acute bacterial enteritis in the United States and many other industrialized countries (*1**,**2*). When the diagnosis of infection is based exclusively on culturing on selective media, >95% of *Campylobacter* infections are caused by *Campylobacter jejuni* or *C. coli*. However, with refinements in isolation and identification methods, other related species such as *C. upsaliensis*, *C. jejuni* subsp. *doylei*, *C. fetus* subsp *fetus*, *C. concisus*, *Arcobacter butzleri*, *Helicobacter fennelliae*, and *H. cinaedi*, have emerged as potential enteric human pathogens (*3*). Since most laboratories do not use appropriate culture conditions to detect all *Campylobacter* spp. and related organisms or do not identify isolates to species level, data on the incidence and clinical importance of these non–*C. jejuni/coli* organisms are scarce.

During the past decade, improvements in isolation techniques in veterinary medicine have led to the discovery of *Arcobacter* spp. as animal pathogens. Members of the genus *Arcobacter* are aerotolerant *Campylobacter*-like organisms. They were first isolated from aborted bovine fetuses by Ellis in 1977 (*4*). *Arcobacter* spp. differ from *Campylobacter* spp. by their ability to grow at lower temperatures and in air (*5*). Later, *Arcobacter* infections in humans were also described. Two species, *A. butzleri* and, more rarely, *A. cryaerophilus*, have been associated with enteritis and occasionally bacteremia (*6**–**9*). Patients with *A. butzleri* infections report diarrhea associated with abdominal pain; nausea and vomiting or fever also occur (*10**,**11*). A third species, *A. skirrowii*, has recently been isolated from a person with chronic diarrhea (*12*). Despite these occasional reports, the contribution of *Arcobacter* species to human diarrhea is still unknown. The aim of our study was to compare the prevalence and the clinical features of *A. butzleri* isolated from stools with those of *C. jejuni*.

## The Study

From January 1995 to December 2002, all stool samples submitted to two hospital laboratories serving the Brugmann, Queen Fabiola, and Saint-Pierre University Hospitals in Brussels, Belgium, were examined macroscopically for consistency, gross blood, and mucus and microscopically for parasites, leukocytes, and erythrocytes. These samples were also cultured for common bacterial pathogens. Stool samples of patients <2 years of age were also evaluated for rotavirus and enteric adenovirus since viral diarrhea is mainly seen in young children.

A specific culture protocol for the recovery of *Campylobacter* spp. and related organisms consisting of one solid selective medium and a filtration method was systematically applied. In the selective agar method, the fecal suspension (approximately 1 g/mL of saline) was plated onto Butzler's medium comprising Mueller-Hinton agar (Oxoid Ltd, Basingstoke, United Kingdom) containing 5% sheep blood and the antimicrobial supplement cefoperazone 30 mg/L, rifampicin 10 mg/L, and amphotericin B 2 mg/L (Institute Virion, Rüschlikon, Switzerland) (*13*). The plates were incubated for 48 h at 42°C in a microaerobic atmosphere (5% O_2_, 6% H_2_, 10% CO_2_, and 79% N_2_). The plates were examined daily for growth of *Campylobacter* species.

The membrane filtration method was performed according to Lopez (*14*). Stool samples were diluted 1:5 in Brucella broth. Cellulose acetate filters (50 mm in diameter) with a pore size of 0.45 µm were placed on the surface of Mueller-Hinton agar plates containing 5% sheep blood. Eight drops of the fecal suspension were placed on the top of the membrane and allowed to filter passively for 30 min at 37°C in air. After filtration, the filters were removed, and the plates were incubated at 37°C in a microaerobic atmosphere for up to 10 days.

Two specific procedures for isolating *Arcobacter* were used successively. Until April 1995, we used a membrane filtration technique (*15*). Subsequently, we switched to direct plating on *Arcobacter* selective medium (*16*), for which 0.5 g of stool samples was injected into an enrichment broth (Brucella broth + antimicrobial supplement consisting of piperacillin 75 mg/L, cefoperazone 25 mg/L, amphotericin B 10 mg/L, trimethoprim 20 mg/L, and cycloheximide 100 mg/L + 5% laked horse blood) and incubated for 24 h at 25°C in a microaerobic atmosphere. After incubation, 40 µL of enriched broth was plated onto *Arcobacter* selective medium (*16*). The plates were then incubated for 3 days at 25°C in a microaerobic atmosphere and examined daily.

Gram-negative, motile, spiral, or curved rods were identified as *Campylobacter* by morphologic and biochemical characteristics consistent with the genus *Campylobacter*, as recommended by Vandamme et al. (*5*). A complementary distinction between *A. butzleri* and *A. cryaerophilus* was achieved by using sodium dodecyl sulfate–polyacrylamide gel electrophoresis of whole-cell proteins and by a multiplex polymerase chain reaction assay (*17*).

To assess the pattern of clinical disease, patients with *A. butzleri* infection were matched against three randomly selected control patients with *C. jejuni* infection. To achieve this, all charts of patients with *A. butzleri* infection and controls were reviewed retrospectively by combining the records of the medical and microbiology departments. A structured, close-ended questionnaire was used to collect the patient's history, age, sex, status (outpatient or hospitalized for >48 h) and history of international travel. The clinical history included diarrhea within the preceding 3 months, duration of symptoms, nature of the diarrhea (watery or bloody and presence of cells), intensity of fever, nausea or vomiting, abdominal pains, asymptomatic carriage (routine screening of enteric pathogens in stools of HIV-infected persons, other immunocompromised states, and patients from a foreign country referred for surgery), and underlying disease. Diarrhea was defined as at least three unformed or liquid stools per day for at least 3 days. Because of the study's retrospective nature, we could only classify diarrhea as acute (duration <15 days) or chronic (duration >15 days). Treatment history included antimicrobial history and use of intravenous fluid therapy, and, finally, clinical status after 1 month of treatment (cure or persistent diarrhea).

All parameters were compared between index patients and matched controls. Comparisons were made by Pearson χ^2^ test for 1:3 control data. Odds ratios (OR) and 95% confidence intervals (CI) were calculated.

From January 1995 to December 2002, a total of 67,599 stool specimens from 40,995 patients were submitted for bacteriologic stool culture. These cultures yielded *Campylobacter* and related organisms in 1,906 patients, *Salmonella* species in 1,720 patients, and *Shigella* species in 244 patients. Other identified pathogens include enteropathogenic *Escherichia coli* (137 patients), *Yersinia enterocolitica* (87 patients), *Plesiomonas* spp. (22 patients), and *Aeromonas* spp. (21 patients).

Among the 1,906 *Campylobacter* and related organisms isolated during the study period, 77.2% were *C. jejuni*, 11.4% were *C. coli*, and 4.5% were *C. upsaliensis* (Table 1). Ninety-seven *Arcobacter* isolates were obtained from 77 patients. Among them, *A. butzleri* was the most frequently isolated species (84 isolates from 67 patients), accounting for 3.5 % of the *Campylobacter* and related organisms bacterial group. Thirteen *A. cryaerophilus* isolates from 10 patients were obtained, but no *A. skirrowii* isolates were found. Other species, such as *C. concisus*, *C. fetus*, *C. curvus*, *C. lari*, and *C. hyointestinalis*, were also found in small numbers (Table 1).

**Table 1:** Distribution of *Campylobacter* spp. and related organisms isolated from 1,906 patients,^a^ January 1995–December 2002, and comparison of recovery by isolation method used^b^

Species	No. (%)	No. of patients positive for *Campylobacter* by one medium or a combination of media						
		BSM	ASM	FM	BSM + FM	BM + ASM	ASM + FM	All methods
*Campylobacter jejuni* subsp. *jejuni*	1,471 (77.2)	1,353	12	1,076	1,471	1,353	1,081	1,471
*C. coli*	218 (11.4)	199	5	174	218	199	174	218
*C. upsaliensis*	85 (4.5)	7	0	85	85	7	85	85
*Arcobacter butzleri*	67 (3.5)^c^	3	65	5	5	65	65	67
*C. concisus*	27 (1.4)	0	0	27	27	0	27	27
*C. fetus* subsp. *fetus*	11 (0.6)	2	0	9	11	2	9	11
*A. cryaerophilus*	10 (0.5)	0	9	1	1	9	10	10
*C. curvus*	9 (0.5)	0	0	9	9	0	9	9
*C. lari I*	3 (0.2)	1	0	3	3	1	3	3
*C. hyointestinalis*	2 (0.1)	0	0	2	2	0	2	2
*Helicobacter pullorum*	2 (0.1)	0	0	2	2	0	2	2
*C. sputorum*	1 (0.1)	0	0	1	1	0	1	1
Total	1,906 (100.0)	1,565	91	1,394	1,835	1,636	1,468	1,906

^a^At the Saint-Pierre, Brugmann, and Queen Fabiola University Hospitals. ^b^BSM, Butzler selective medium; ASM, *Arcobacter* selective medium; FM, filtration method. ^c^Two of the 67 *A. butzleri* isolated were recovered by the filtration method (*15*) in use up to April 1995.

Medical records were available for 61 of the 67 patients with *A. butzleri* infection. Ages of patients with *A. butzleri* infection were 30 days–90 years; there were slightly more female than male patients. Fourteen patients were hospitalized for >48 h. Four patients had traveled abroad before onset of symptoms. Ten patients had underlying disease: 4 were HIV seropositive, and 3 were immunocompromised (postrenal graft, celiac disease, and chemotherapy for cerebellar astrocytoma). Other chronic illnesses included dementia (n = 1), insulin-dependent diabetes mellitus (n = 1), and hepatitis C (n = 1).

Thirty-one patients complained of acute diarrhea (>24 watery stools) lasting for 3 to 15 days, and 10 had persistent or recurrent diarrhea lasting >2 weeks–2 months. Six patients had abdominal pain without diarrhea. Twelve patients were asymptomatic. Sixteen patients received antimicrobial therapy, but only 7 were treated empirically with an antimicrobial agent for which the strain was susceptible. The symptoms resolved in all patients except one, regardless of the antimicrobial agent used. Among patients treated symptomatically, three patients had persistent or recurrent symptoms.

Sixty-seven patients with *A. butzleri* infection were matched against 201 patients with *C. jejuni* infection. The age and sex distributions were similar for patients colonized by each species. No significant differences in international travel were observed. However, proportionally fewer patients with *A. butzleri* (79.1%) were treated as outpatients than those with *C. jejuni* (90.5%) (OR 0.40, 95% CI 0.17–0.90) (Table 2).

**Table 2:** Case-control study of microbiologic and clinical features of patients with *Arcobacter butzleri* and *Campylobacter jejuni* infection

Features/treatment/outcome	No. patients with *A. butzleri* infection (%)	No. patients with *C. jejuni* infection (%)	OR (95% CI)
Microbiologic features			
Erythrocytes in stool	4/67 (6.0)	59/201 (29.4)	0.15 (0.05–0.46)
Leukocytes in stool	2/67 (3.0)	36/201 (17.9)	0.14 (0.02–0.62)
Associated organisms	12/67 (17.9)	24/201 (11.9)	1.61 (0.71–3.63)
Clinical features			
Diarrhea	41/61 (67.2)	149/191 (78.0)	0.58 (0.29–1.14)
Acute diarrhea	31/61 (50.8)	140/191 (73.3)	0.38 (0.20–0.79)
Persistent diarrhea	10/61 (16.4)	9/191 (4.7)	3.97 (1.40–11.33)
Watery diarrhea	31/61 (50.8)	61/191 (31.9)	2.20 (1.18–4.13)
Fever, temperature >38.5°C	20/61 (32.8)	83/191 (43.5)	0.63 (0.33–1.21)
Nausea, vomiting, or both	17/61 (27.9)	47/191 (24.6)	1.18 (0.59–2.37)
Abdominal pain	18/61 (29.5)	53/191 (27.7)	1.09 (0.55–2.15)
Asymptomatic carriage	12/61 (19.7)	19/191 (9.9)	2.22 (0.94–5.21)
Underlying disease	10/61 (16.4)	49/191 (25.7)	0.57 (0.25–1.27)
Treatment			
Antimicrobial agents	16/61 (26.2)	79/191 (41.4)	0.50 (0.25–1.00)
Intravenous fluid therapy	1/61 (1.6)	8/191 (4.2)	0.38 (0.38–3.05)
Outcome			
Relapse	4/61 (6.6)	6/191 (3.1)	2.16 (0.49–9.06)

^a^OR, odds ratio; CI, confidence interval.

Rectal bleeding, inflammatory exudates, or both were significantly less common in *A. butzleri* than in *C. jejuni* infection (OR 0.15, 95% CI 0.05–0.46). A concomitant infection with another enteric pathogen occurred in a higher proportion of patients with *A. butzleri* infection than patients with *C. jejuni* infection, but the difference was not significant. Twelve patients with *A. butzleri* infection had a coinfection with one of the following enteric pathogens: *Salmonella enterica* (n = 4) (2 ser. Enteritidis, 1 ser. Typhimurium and 1 ser. Virchow), *Rotavirus* (n = 3), *C. jejuni* (n = 2), *Giardia lamblia* (n = 2), and *Clostridium difficile* toxin B positive (n = 1).

In 24 patients with *Campylobacter jejuni* infection, we found a coinfection with one another enteric pathogen: *S. enterica* (*7*) (3 ser. Enteritidis, 3 ser. Typhimurium, and 1 ser.Virchow), *Rotavirus* (*5*), *Adenovirus* (*4*), *Giardia lamblia* (*2*), *Shigella flexneri* (*2*), *Yersinia enterocolitica* (*1*), and *Clostridium difficile* toxin B positive (*1*). In two additional cases of campylobacteriosis, we found a coinfection with two other enteric organisms: one patient was infected with *S. dysenteriae* and *Hymenolepis nana* and the other patient with *Rotavirus* and *Salmonella enterica* serotype Typhimurium.

To assess the prevalence of *A. butzleri* and *Campylobacter jejuni* in diarrheic stool specimens, we considered only stools with loose or liquid macroscopic aspect as diarrhea. Among the 67,599 stool specimens received, 12,413 were solid stools, and 55,186 were diarrheic. Among the diarrheic stool specimens, we isolated *A. butzleri* and *C. jejuni* in 77 (0.14%) and 3,209 (5.81%) stools, respectively. Among the nondiarrheic stool specimens, we isolated *A. butzleri* and *C. jejuni* in 7 (0.06%) and 205 (1.65%) stools, respectively. *A. butzleri* was more frequently isolated from diarrheic stool specimens than from nondiarrheic stools specimens. This difference was significant (OR 2.48, 95% CI 1.10–5.86) (p = 0.0175). We observed a similar result for the recovery of *C. jejuni* from diarrheic stool specimens compared with nondiarrheic stools specimens. This difference was even more significant (OR 3.68, 95% CI 3.18–4.25) (p < 0.0001).

Because medical records were unavailable for 6 patients with *A. butzleri* and 10 patients with *C. jejuni*, we compared the clinical features of 61 patients with *A. butzleri* matched against those of 191 patients with *C. jejuni*. Although diarrhea was a common clinical feature of both groups, it was more frequent in the *C. jejuni–*infected patients. The characteristics of the diarrhea differed significantly, however. Patients with *A. butzleri* were more likely to have persistent diarrhea (OR 3.97, 95% CI 1.4–11.3), or watery diarrhea (OR 2.20, 95% CI 1.18–4.13) than those with *C. jejuni* infection, but they were less likely to have acute diarrhea (OR 0.38 95%, CI 0.20–0.79). Other clinical features did not differ significantly. Asymptomatic carriage was more frequently encountered in patients with *A. butzleri* infection than in those with *C. jejuni* infection, but not significantly (OR 2.22, 95% CI 0.94–5.21).

Proportionally more patients with *C. jejuni* infections were treated with antimicrobial agents (41.4%) than patients with *A. butzleri* infections (26.2%) (OR 0.50, 95% CI 0.25–1.0). Among them, only 43.8% of patients with *A. butzleri* infections were treated empirically with an antimicrobial agent for which the strain was susceptible, whereas 79.5% of patients with *C. jejuni* received an appropriate antimicrobial drug.

## Conclusions

In this study, *Arcobacter* was the fourth most common *Campylobacter* or *Campylobacter*-like organism isolated from stool specimens in our laboratories. Our observations suggest that *A. butzleri* display similar microbiologic and clinical features as *C. jejuni*. However, compared with *C. jejuni*, *A. butzleri* are more frequently associated with a persistent and watery diarrhea and less associated with bloody diarrhea. This first study on *Arcobacter* in humans could be the beginning of future research to better understand the pathogenesis and epidemiologyof these non–*jejuni/coli Campylobacter*.
